# Supplementary material for: Bacteriophage-driven emergence and expansion of Staphylococcus aureus in rodent populations
Source: PLoS Pathog. 2024 Jul 24;20(7):e1012378. doi: 10.1371/journal.ppat.1012378 (PMC11299810; doi:10.1371/journal.ppat.1012378)
Supplement: S1 Fig — The plot shows all possible topologies, parts of the tree with a higher topology agreement look sharper, whereas areas with more uncertain topologies look more blurred. (DOCX) [file ppat.1012378.s001.docx]

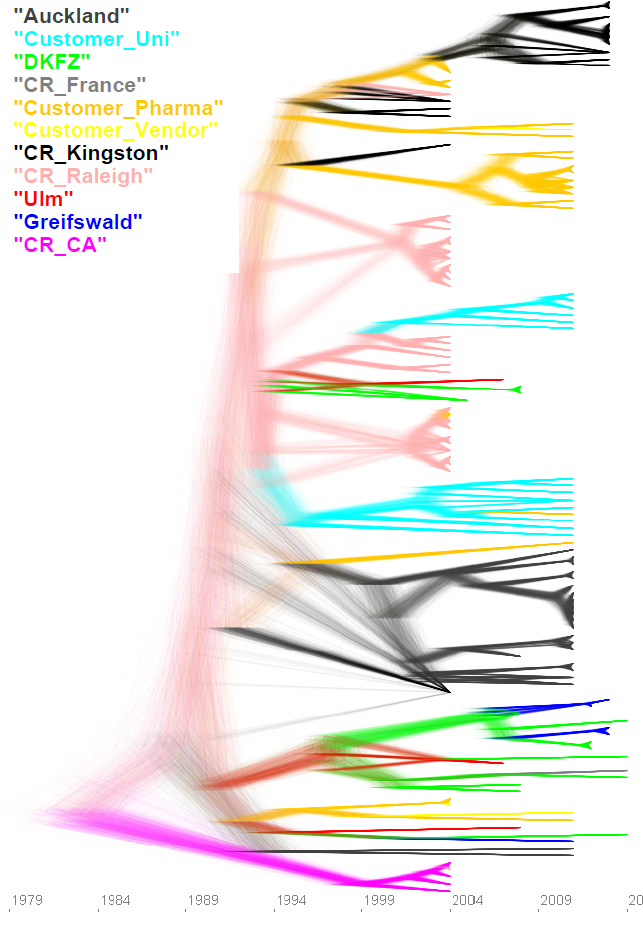


**S1 Fig.** Densitree of Bayesian posterior distribution of CC88 international murine cluster. The plot shows all possible topologies, parts of the tree with a higher topology agreement look sharper, whereas areas with more uncertain topologies look more blurred.
