# Supplementary material for: Bacteriophage-driven emergence and expansion of Staphylococcus aureus in rodent populations
Source: PLoS Pathog. 2024 Jul 24;20(7):e1012378. doi: 10.1371/journal.ppat.1012378 (PMC11299810; doi:10.1371/journal.ppat.1012378)
Supplement: S2 Fig — (DOCX) [file ppat.1012378.s002.docx]

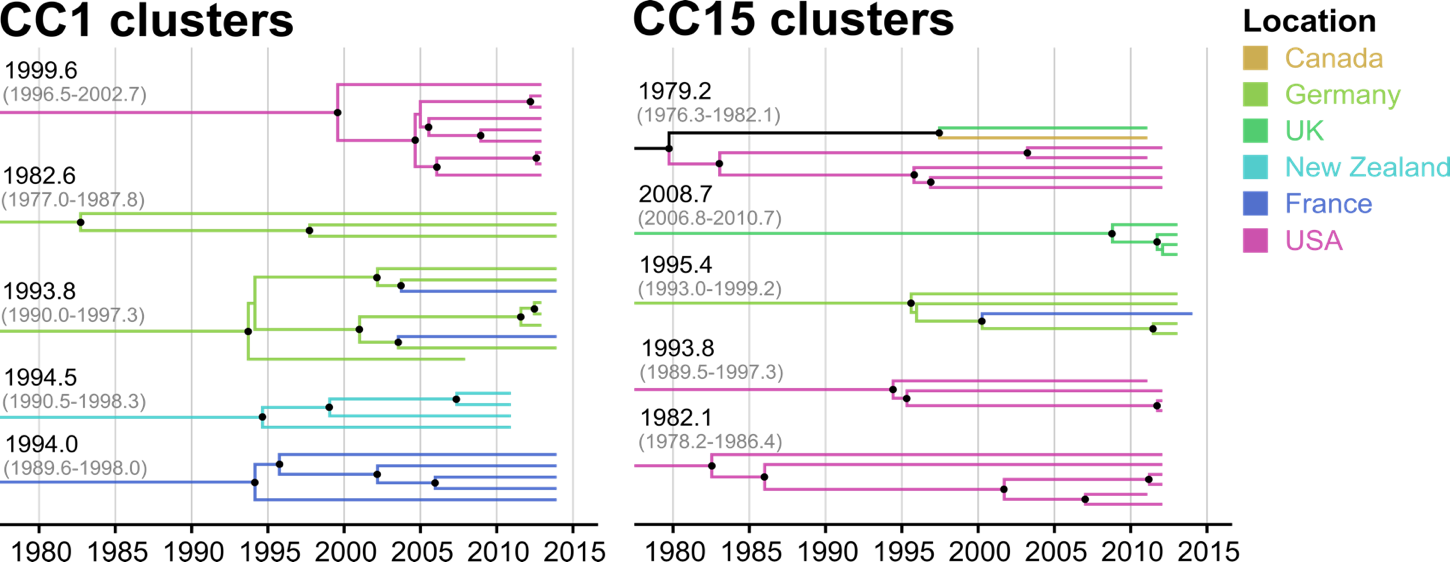


**S2 Fig**. CC1 and CC15 murine-specific clusters extracted from the phylogenetic Bayesian trees of both *S. aureus* lineages.
