# Supplementary material for: Bacteriophage-driven emergence and expansion of Staphylococcus aureus in rodent populations
Source: PLoS Pathog. 2024 Jul 24;20(7):e1012378. doi: 10.1371/journal.ppat.1012378 (PMC11299810; doi:10.1371/journal.ppat.1012378)
Supplement: S3 Fig — Tips names and circles are coloured according to the integrase type detected through BLAST. (DOCX) [file ppat.1012378.s003.docx]

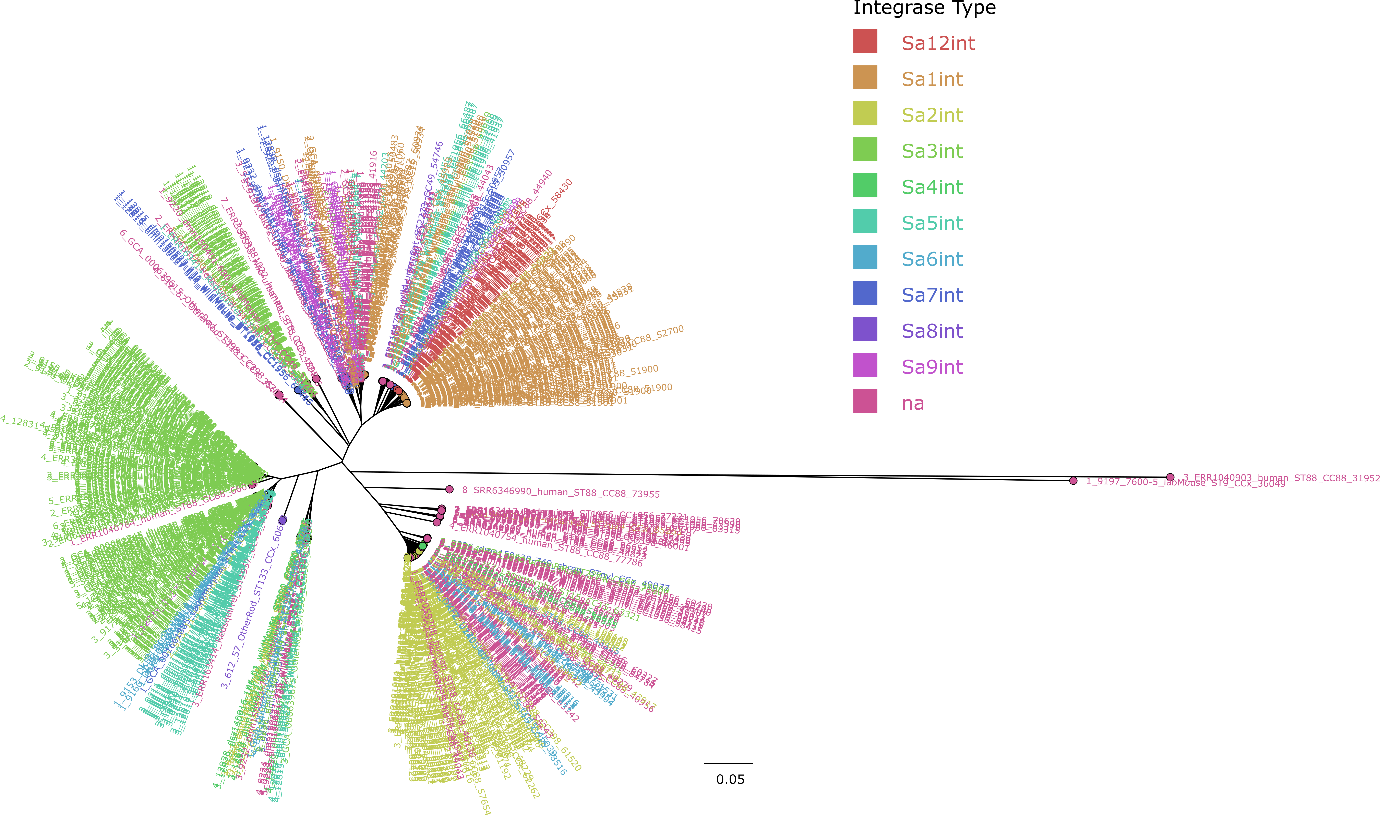


**S3 Fig**. Hierarchical clustering tree built from pairwise genetic distances (calculated with Mash) between prophage sequences (as predicted by PHASTER) across the whole study dataset. Tips names and circles are coloured according to the integrase type detected through BLAST.
