# Supplementary material for: Bacteriophage-driven emergence and expansion of Staphylococcus aureus in rodent populations
Source: PLoS Pathog. 2024 Jul 24;20(7):e1012378. doi: 10.1371/journal.ppat.1012378 (PMC11299810; doi:10.1371/journal.ppat.1012378)
Supplement: S4 Fig — Reference sequences for each integrase type are coloured in cyan. Putative novel integrase types, genetically different and showing different integration sites, are coloured in orange. (DOCX) [file ppat.1012378.s004.docx]

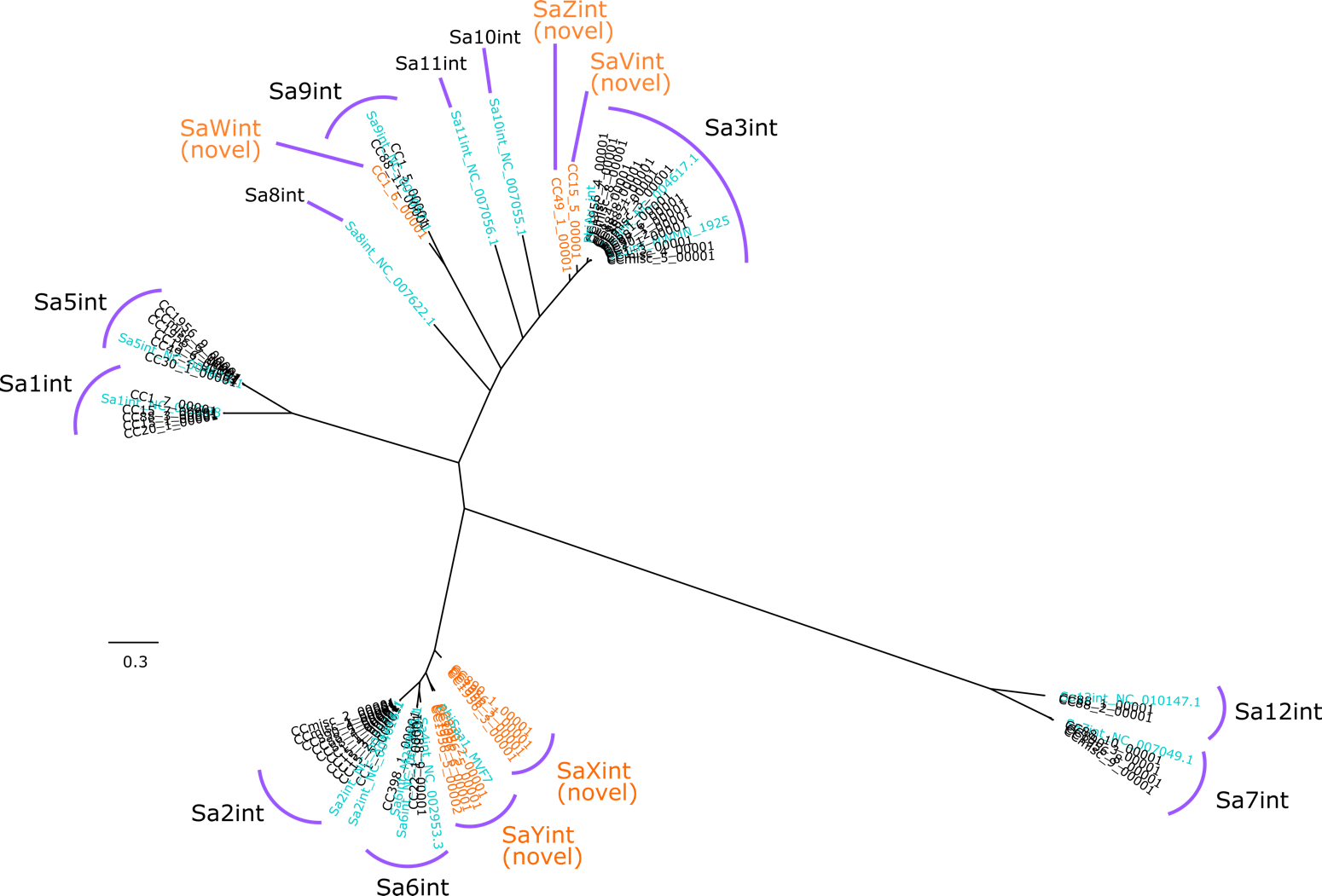


**S4 Fig**. Maximum-likelihood phylogenetic analysis of the integrases in rodent prophages. Reference sequences for each integrase type are coloured in cyan. Putative novel integrase types, genetically different and showing different integration sites, are coloured in orange.
