# Supplementary material for: Bacteriophage-driven emergence and expansion of Staphylococcus aureus in rodent populations
Source: PLoS Pathog. 2024 Jul 24;20(7):e1012378. doi: 10.1371/journal.ppat.1012378 (PMC11299810; doi:10.1371/journal.ppat.1012378)
Supplement: S5 Fig — A) Amino acid-based identity matrix for core-genome encoded coa (human-derived ST8, laboratory mouse-derived ST88, and bank vole-derived ST49 strains) as well as phage-encoded vWbp variants from yellow-necked field mice (Coa’ ST980) and common voles (Coa’ ST3252). B) Alignment of core genome- and phage-encoded coagulases. The prothrombin-binding domains D1 and D2 and the fibrinogen-binding domain are indicated. Amino acid residues interacting with the exosite 1 on prothrombin are highlighted. The highlighted N-terminal amino acids induce allosteric activation of prothrombin by inserting into the prothrombin activation pocket. C) Amino acid-based identity matrix for core-genome encoded vWbp (human-derived ST8, laboratory mouse-derived ST88, and bank vole-derived ST49 strains), as well as SaPI-endoded vWbp’ (common shrew-derived ST3033). D) Alignment of core genome- and SaPI-encoded vWbps.The N-terminal peptide, the prothrombin-binding domains D1 and D2 and the van Willebrand factor-binding domain are indicated. (DOCX) [file ppat.1012378.s005.docx]

**
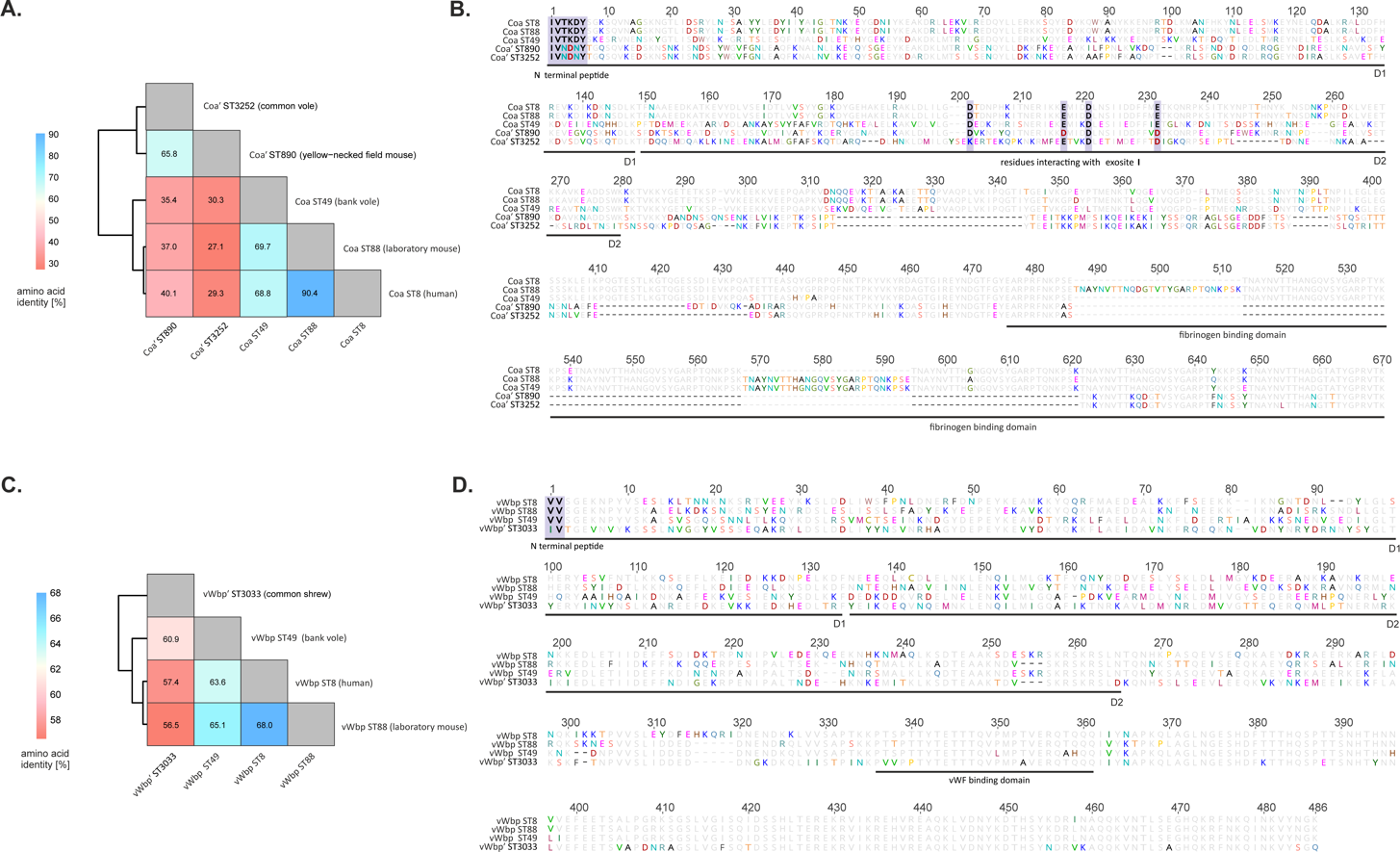
**

**S5 Fig.** Protein sequence variation among core genome- and MGE-encoded coagulases. A) Amino acid-based identity matrix for core-genome encoded coa (human-derived ST8, laboratory mouse-derived ST88, and bank vole-derived ST49 strains) as well as phage-encoded vWbp variants from yellow-necked field mice (Coa’ ST980) and common voles (Coa’ ST3252). B) Alignment of core genome- and phage-encoded coagulases. The prothrombin-binding domains D1 and D2 and the fibrinogen-binding domain are indicated. Amino acid residues interacting with the exosite 1 on prothrombin are highlighted. The highlighted N-terminal amino acids induce allosteric activation of prothrombin by inserting into the prothrombin activation pocket. C) Amino acid-based identity matrix for core-genome encoded vWbp (human-derived ST8, laboratory mouse-derived ST88, and bank vole-derived ST49 strains), as well as SaPI-endoded vWbp’ (common shrew-derived ST3033). D) Alignment of core genome- and SaPI-encoded vWbps.The N-terminal peptide, the prothrombin-binding domains D1 and D2 and the van Willebrand factor-binding domain are indicated.
