# Supplementary material for: Bacteriophage-driven emergence and expansion of Staphylococcus aureus in rodent populations
Source: PLoS Pathog. 2024 Jul 24;20(7):e1012378. doi: 10.1371/journal.ppat.1012378 (PMC11299810; doi:10.1371/journal.ppat.1012378)
Supplement: S6 Fig — A) Amino acid-based identity matrix for the prothrombin heavy chain derived from humans, laboratory mice, yellow-necked field mice, rats, bank voles and common voles. B) Alignment of human, rodent and shrew prothrombin sequences (heavy chain). The two Coagulase binding sites, Trp148 binding pocket and exosite 1, are indicated. Both Coa and vWbp allosterically activate prothrombin through insertion of their first two N-terminal residues into activation pocket on prothrombin. (DOCX) [file ppat.1012378.s006.docx]

**
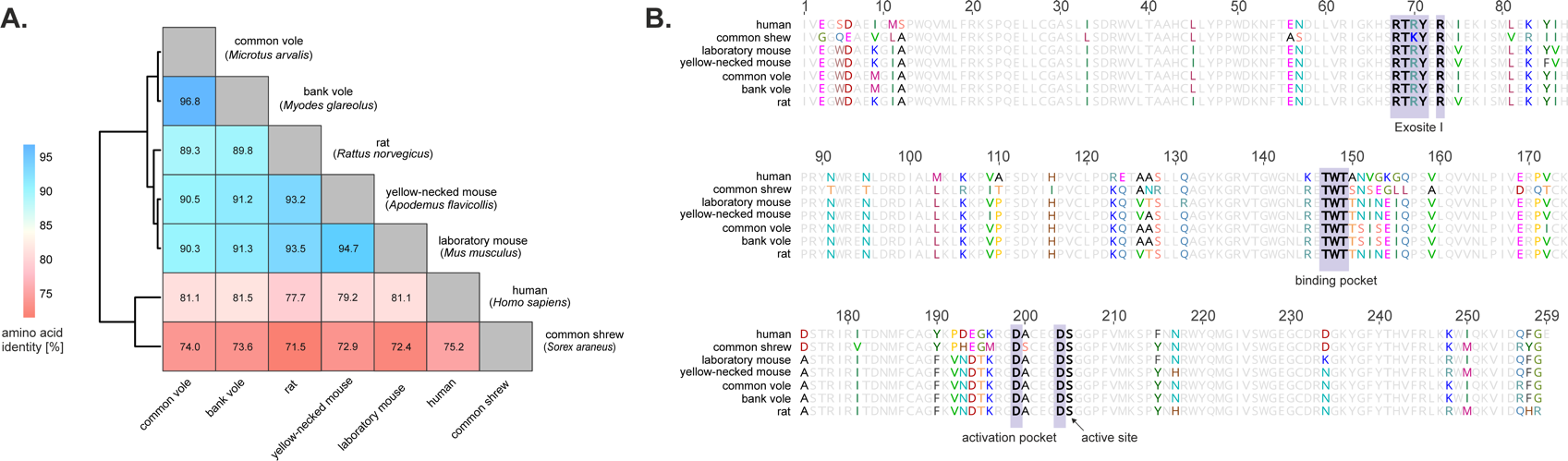
**

**S6 Fig.** Protein sequence variation among human, rodent and shrew prothrombin. A) Amino acid-based identity matrix for the prothrombin heavy chain derived from humans, laboratory mice, yellow-necked field mice, rats, bank voles and common voles. B) Alignment of human, rodent and shrew prothrombin sequences (heavy chain). The two Coagulase binding sites, Trp148 binding pocket and exosite 1, are indicated. Both Coa and vWbp allosterically activate prothrombin through insertion of their first two N-terminal residues into activation pocket on prothrombin.
