# Supplementary material for: Bacteriophage-driven emergence and expansion of Staphylococcus aureus in rodent populations
Source: PLoS Pathog. 2024 Jul 24;20(7):e1012378. doi: 10.1371/journal.ppat.1012378 (PMC11299810; doi:10.1371/journal.ppat.1012378)
Supplement: Attachment — Submitted filename: Murine Staph_Response to reviewers.docx [file ppat.1012378.s013.docx]

We are grateful to the editor and reviewers for their constructive comments. We have carefully considered each of the points made and addressed them as indicated below in blue text.

**Editor:**

In addition, the posterior probabilities are not high in some of your phylogeographic and phylogenetic analyses, which needs to be better acknowledged. For example, analysis in Figure 2A may best support an origin of CC88 in SE Asia, but it does so at only 32% PP - hardly convincing. When looking at the geographic origin of murine CC88 in Figure 2A the pie-chart seems to only support Canada at about 50% PP, but in the text describing Figure 2B you indicate the support is 86% (probably should also show pie-charts in 2B).

The Figure 2B result is better, but why the difference? Is it sampling related? Based on the analysis presented, you should be more circumspect in the Abstract about where this murine lineage may have emerged.

The posterior probability value of 50% was obtained in the phylogeographic analysis using countries/geographical regions (Figure 2A), whereas PP=86% is the result in the analysis for the rodent CC88 cluster that uses specific laboratory locations (Figure 2B). These results were expected to be different as they use distinct sequence data and location states.

We have adjusted the abstract as suggested and the pie charts are now included for 2 key nodes in Fig 2B.

In Figure 3, the CC49 node that includes human and rodent isolates has weak support, possibly due to a closely timed radiation of lineages?

We agree that this is a likely explanation.

**Part I - Summary**

Reviewer #1: 
The only minor comment is that it may be worth mentioning that the limited geographic sampling of wild mouse, vole, shrew, and guinea pig isolates limits the phylogeographic analysis (i.e., you can’t infer ancestral locations from unsampled locations). However, the discussion mentions something to this effect.

We are grateful for the comment and have now added this aspect to the discussion (P.17 line 22-23)

In supplemental figure 3, you could probably drop the tip labels and just use shapes since you can’t read the text, but again, that is a minor stylistic critique.

We are grateful for the suggestion but have respectfully left the tip labels which help to give a sense of the number of taxa in each clade.

**Part II – Major Issues: Key Experiments Required for Acceptance**

Reviewer #2: 1. Sampling is key in this kind of studies, yet little is said on how the isolates were collected. How were they obtained? Were they selected somehow?

In 2013, we reported for the first time that laboratory mice can be colonized with *S. aureus* isolates (Holtfreter et al., PLoSOne 2013). Over the next years, we collected 230 murine S. aureus isolates from various vendors (Charles River, US; Charles River, Canada; Charles River, France, an anonymous US vendor), four research institutes (University of Auckland, New Zealand; University Medicine of Greifswald, Germany; DKFZ, Germany; University of Ulm, Germany) and a pharmaceutical company (Beijing, China) (detailed in Mrochen et al, Int J Medical Microbiol 2018). Sampling was not performed systematically, but based on existing (e.g. Charles River) or newly formed collaborations (Ulm, DKFZ), Beijing), the latter ones sparked by our 2013 report. Specimen were obtained during routine health monitoring, and occasionally from euthanized mice suffering from *S. aureus* infections (e.g. preputial gland adenitis). To avoid a sampling bias, we included a maximum of 4 isolates of the same *spa* type, barrier, mouse strain and time point in the study cohort.

We have now added this information in the methods section (P.4 line 26-30.

2. Multiple GWAS approaches were used (fixed effect models, linear mixed model, lineage effects), yet it is unclear how the output of the approaches overlaps (or not) and how the final 41 hits were selected (i.e. just one approach? Intersection of multiples approaches?). I recommend providing all GWAS results in the supplementary data, including non-significant hits.

Even though we did assess different combinations of models, we selected the results from the GWAS analysis using *mash* distances for population structure correction and the fixed effects association model, as this combination was less likely to be confounded by clade-specific hits (as demonstrated by initial Manhattan plots which indicated groups of GWAS hits with the same probability).

We now provide the full GWAS results as a supplementary data file as suggested (“Supp_data_full_gwas_results_pyseer.csv”).

**Part III – Minor Issues: Editorial and Data Presentation Modifications**

Reviewer #1: Minor changes to supplemental figures, but not required

Reviewer #2: 1. Please add line numbers to the manuscript

Now done.
2. Please provide the heritability estimated calculated by Pyseer

The heritability calculated by pyseer was h^2^ = 0.78. However, this calculation was only performed using the mixed effects model (*FaST-LMM*) which was not included in the final analyses.

3. From figure 4B it looks like the GWAS was done on a per-CC basis, however this is not clearly said in the results: please clarify.

The GWAS was performed using all data, but because of the genetic diversity of the data, there was no one given reference genome to which all resulting hits could be mapped. For illustration purposes, 4 individual Manhattan plots were generated, each showing hits mapped to a representative reference genome of the major rodent CCs included in the analysis.

4. Table S1: please list all genomes included in the analysis

We have added a list of all genomes used in the phylogenetic analyses in a new supplementary Table S1.

5. “Overall, the relatively small number of GWAS hits out-with MGE indicates that convergent adaptations of different S. aureus lineages to the rodent host were rare”: this sentence is unclear to me.

We thank the reviewer for pointing out this lack of clarity and we have rewritten the sentence (P.18 line 6-8).

 We thank the editor and reviewers again for their supportive comments. Please don’t hestitate to contact us if you require any further clarification.
